# *In loco* provision of physical therapy services to military firefighters involved in Brumadinho dam disaster

**DOI:** 10.31744/einstein_journal/2022AO5885

**Published:** 2022-02-03

**Authors:** Marcelo von Sperling de Souza, Anna Florence Alves Paulino de Souza, Pollyanna Figueiredo Gomes, Bárbara Brito de Carvalho e Borges, Roseane Marques Ribeiro, Maria Rosália de Faria Moraes

**Affiliations:** 1 Hospital da Polícia Militar Belo Horizonte MG Brazil Hospital da Polícia Militar, Belo Horizonte, MG, Brazil.

**Keywords:** Natural disasters, Physical therapy specialty, Military health, Disaster team, Musculoskeletal pain

## Abstract

**Objective:**

To investigate the impact of *in loco* physical therapy interventions on military firefighters involved in search and rescue operations following the collapse of the Brumadinho dam, in Minas Gerais. To describe the clinical and demographic profile of military firefighters receiving physical therapy care.

**Methods:**

Physical therapy assessment and care protocols were designed. Protocols were based on manual physical therapy approaches, and aimed primarily to alleviate musculoskeletal pain. Physical therapists involved were duly trained prior to interventions to level technical skills. Physical therapy was provided upon request (*i.e*., military workers sought the service after work shift completion).

**Results:**

A total of 318 military firefighters, most of whom were males (92.5%) mean age 32.9 years, received physical therapy care (575 sessions spread out over 48 days). In this sample, 43.4% of military workers had a history of musculoskeletal complaints. Military workers seeking physical therapy after completion of their work shifts reported mean pain intensity of 5.4 in a numerical scale. Spinal pain was reported in 61.7% of cases, followed by generalized muscle and myofascial pain (16.7%), lower and upper limb pain (14.4% and 6.8%, respectively). At the end of sessions, mean pain intensity reported dropped down to 1.3. Differences were statistically significant (non-parametric Wilcoxon test; p=0.001).

**Conclusion:**

The unprecedented physical therapy intervention described had a positive impact on relief of musculoskeletal pain among military firefighters involved in search and rescue operations during the Brumadinho dam disaster, and seen at the end of their work shifts.

## INTRODUCTION

On January 25, 2019, *Mina Córrego do Feijão* dam collapsed. This dam belonged to the mining company *Vale S.A.*, located in the municipality of Brumadinho, in the metropolitan region of Belo Horizonte, Minas Gerais. The avalanche of mining waste unleashed by this collapse caused one of the worst environmental and humanitarian disasters in the history of Brazil. As of March 2020, 259 deaths had been officially confirmed and 11 people were missing.^(1)^

Public security forces were called upon at different levels to respond to the disaster. Search and rescue operations were coordinated by *Corpo de Bombeiros Militar de Minas Gerais* (CBMMG) [Military Firefighters Brigade] and supported by troops from several Brazilian states and Israeli military forces. Long lasting and physically demanding operations led to musculoskeletal system overload among military workers involved, with increased risk of dysfunction. Hence, a joint initiative between physical therapists employed by *Polícia Militar de Minas Gerais* (PMMG) [Military Police of Minas Gerais State], *Assessoria de Saúde do CBMMG* [Health Department of CBMMG], *Conselho Regional de Fisioterapia e Terapia Ocupacional da 4ª Região* (Crefito-4) [Regional Physical Therapy and Occupational Therapy Council – 4^th^ Region], and *Sociedade Nacional de Fisioterapia Esportiva-Regional Minas Gerais* (SONAFE-MG) [National Society of Sports Physical Therapy – Minas Gerais Regional Office] was designed to manage and provide physical therapy services to military firefighters.

On February 10, 2019, military firefighters began to receive in-person physical therapy. Care was provided by two teams based at different locations: Clube Aurora, in the municipality of Brumadinho, and the rural neighborhood of Córrego do Feijão. Teams comprised one military and one volunteer civil physical therapist. A total of 19 PMMG and 26 civil physical therapists were involved. Physical therapy team members worked non-stop for 48 days. Other physical therapists were then hired by *Vale S.A*. to continue to provide care to military firefighters.

The role of physical therapists in emergency response to natural disasters has not been well established.^(2,3)^ Scientific publications have shown physical therapists work primarily in multidisciplinary health care teams, providing urgency and emergency care to disaster victims.^(4-7)^ Findings of a literature review conducted by authors of this study suggested physical therapy provision to military workers involved in search and rescue operations was a unique effort, both in the national and the international scenarios.

The pioneer nature of such actions justified this quasi-experimental, retrospective study, aimed to investigate immediate impacts of physical therapy interventions on musculoskeletal pain scores reported by firefighters, after completion of their work shifts. We hypothesized physical therapy interventions would reduce musculoskeletal pain intensity reported by military firefighters after completion of their work shifts. The clinical and demographic profile of firefighters assisted by the physical therapy team was described. Findings of this study may contribute to future research into the impact of physical therapy on occupational health issues among firefighters involved in responses to natural disasters and similar events, as well as to the design of physical therapy support strategies.

## OBJECTIVE

To investigate the impact of *in loco* physical therapy interventions on military firefighters involved in search and rescue operations, following the collapse of the Brumadinho dam, in Minas Gerais.

## METHODS

Assessment and intervention protocols were designed prior to *in loco* interventions. These were based on models of immediate physical therapy services provision to athletes and adapted to activities carried out by firefighters in the specific scenario in question (*i.e*., prolonged physical exertion on unstable surfaces and under extreme conditions). In order to provide a theoretical basis, the Latin American and Caribbean Health Sciences Literature (LILACS) and MEDLINE^®^ databases were searched. Terms used in LILACS database search were “*desastres naturais*”, “*fisioterapia*”, “*atendimento imediato*” and “*dor musculoesquelética*”. The English equivalent terms (“natural disasters”, “physical therapy”, “acute injury management” and “musculoskeletal pain”) were used in MEDLINE^®^ database search. Articles published in English and Portuguese were selected. Scientific data on actions associated with catastrophe management are scarce. Therefore, in an effort to retrieve the largest possible number of studies, the time of search was not restricted.

Technical aspects pertaining to applicable physical therapy interventions were discussed during in-person meetings and interventions designed according to the level of education and expertise of each team member (Annex 1). Major interventions were as follows: manual therapy techniques (mobilization and/or manipulation), manual or instrument-assisted inhibition of trigger points, manual or instrument-assisted myofascial release techniques, and functional bandaging. Daily, non-stop assistance schedules were created.

In-person care was provided at the health care facility deployed at the *Base de Comando e Operações* [Command and Operations Base], in Brumadinho. A dedicated container was used for physical therapy services. This container accommodated two stretchers and materials, such as suction cups, soft tissue mobilization instrument kits, and ethylene-vinyl acetate (EVA) foam rollers for myofascial release and physical therapy exercises.

This quasi-experimental retrospective study was based on data extracted from physical therapy records. The experimental period totaled 48 days, from February 10 to March 29, 2019. Convenience sampling was used. The sample comprised military firefighters directly or indirectly involved in search and rescue operations, who sought physical therapy due to musculoskeletal complaints, after completing their work shift.

Personal information, chief complaint, and self-reported pain intensity (verbal numerical rating scale, VNRS) at the start and end of treatment, as well as main techniques and physical therapy resources employed, were recorded. Data collection duties were assigned to assistant physical therapists during the action planning phase. The following pieces of data were extracted from records: number of military firefighters receiving care, number of physical therapy sessions, age, sex, federal state of origin, affected body segment, primary type of work performed in mission, past medical history of musculoskeletal complaints, and intensity of pain (VNRS score) associated with the cheif complaint at the start and end of treatment.

Data were extracted and entered into an electronic spreadsheet by three independent researchers for statistical analysis, using SPSS, version 11.0.1. Researchers received prior in-person training for data categorization and entry, in order to enhance the homogeneity of procedures. In this phase of the study, missing data or data incorrectly described in records were treated as losses. Lost data were numerically codified in spreadsheets in a standardized manner for appropriate statistical treatment.

Since the same patient could present with pain at different anatomical sites and of varying intensities at different appointments, the variables “chief complaint” (anatomical site of pain) and “pain intensity” were recorded and extracted per session. Self-reported pain intensity was rated using a verbal numerical rating scale, as follows: “Rate the intensity of your pain at this very moment on a zero (no pain at all) to ten (worst pain ever experienced or imaginable) scale”. In patients with multiple complaints, pain intensity was used to determine the site associated with the primary complaint (*i.e*., body segment with the highest VNRS score). In cases with identical pain scores, the site in which pain was more recurrent over the course of sessions was defined as chief complaint for data extraction purposes.

Descriptive statistics were used for sample characterization. Inferential statistics were used for intergroup comparisons and correlations. Missing data were automatically detected and informed separately for each dependent variable. The level of significance was set at 0.05.

Given the retrospective nature of the study, a waiver of Informed Consent Form request was submitted to the Research Ethics Committee. A Term of Commitment to Responsible Data Use (TCUD) was used instead. This term and procedures involved in this research protocol were approved by the Ethics Committee for Research with Human Beings of the *Hospital da Polícia Militar de Minas Gerais*, opinion # 03/2019 and officially approved the Research Ethics Committee of *Secretaria Municipal de Saúde de Belo Horizonte* (SMSA-BH) [Municipal Health Authority], via *Plataforma Brasil*, opinion # 3.682.345 and CAAE: 21336619.8.0000.5140.

## RESULTS

The physical therapy team assigned to Brumadinho provided care to 318 military firefighters, most of whom were males (294 participants, 92.5% of sample). Mean age was 32.9 years (age range, 21 to 53 years; standard deviation, 6.3 years). The relative frequencies of military worker age, distributed according to age ranges are shown in table 1.

**Table 1:** Relative frequency of military workers receiving physical therapy care per age range

Age, years	%
21-23	3.1
24-26	9.3
27-29	21.7
30-32	18.3
33-35	14.1
36-38	15.1
39-41	6.2
42-44	5.8
45-47	4.1
48-50	0.6
51-53	1.7

Physical therapy care was provided upon request (*i.e*., military workers sought the service of their own free will, after completing their work shifts). The number of sessions totaled up 575. The number of sessions per military worker ranged from one to 11 (mean, 1.97; standard deviation, 1.7).

**Figure 1:**
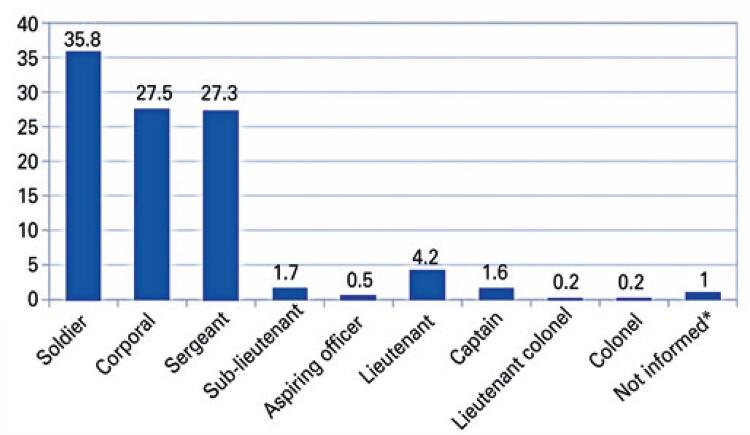
Military worker ranks during Brumadinho (MG) operations

Military firefighter ranks during Brumadinho operations are shown in figure 1. Most military workers in this sample were soldiers (35.8%), followed by corporals (27.5%) and sergeants (27.3%).

* corresponds to six military workers with missing rank data in records.

**Figure 2:**
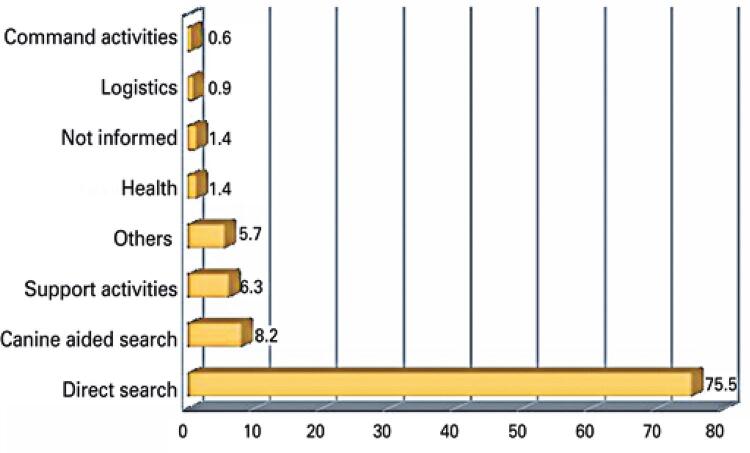
Major role played by military workers involved in Brumadinho (MG) operations

Most military workers (75.5%) were directly involved in search and rescue operations, followed by similar types of operations assisted by dogs (8.2%). The third most common role was operational support (6.3%). Role description was missing in 1.4% of records. In 5.7% of cases, roles were described as “others” (*i.e*., the role reported did not match predetermined categories). These data are shown in figure 2.

In their first appointment, 43.4% of military workers in this sample reported pre-existing musculoskeletal injuries or conditions, whereas 55% did not. These data failed to be recorded in 1.6% of appointments.

The spine was the most common primary site of pain (61.7%), followed by generalized muscle and myofascial pain (16.7%), lower and upper limb pain (14.4% and 6.8%, respectively). These data failed to be recorded in two physical therapy sessions and were therefore categorized as missing data (0.3%) (Table 2).

**Table 2:** Primary site of pain among military workers receiving physical therapy

Anatomical site of pain	Number of physical therapy sessions n (%)
Spine (cervical, thoracic, lumbosacral)	355 (61.7)
Generalized muscle and/or myofascial pain	96 (16.7)
Lower limbs	83 (14.4)
Upper limbs	39 (6.8)
Missing data*	2 (0.3)

* Correspond to two physical therapy sessions in which the site of pain failed to be recorded.

Descriptive pain intensity data are given in table 3. Pain intensity data were missing in 31 and 55 cases (start and end of physical therapy sessions, respectively). Initial and final pain intensity data were available in 517 cases (valid cases, Table 3).

**Table 3:** Pain intensity according to a Verbal Numerical Pain Rating Scale

Pain intensity	n	Minimum value	Maximum value	Mean	Standard deviation
Pain intensity score at the start of session	544	0	10	5.43	1.71
Pain intensity score at the end of session	520	0	8	1.30	1.45
Valid cases	517	-	-	-	-

Zero-to-ten scale where ten designates the worst imaginable pain. Valid cases: number of sessions in which initial and final pain scores were duly measured and recorded.

**Figure 3:**
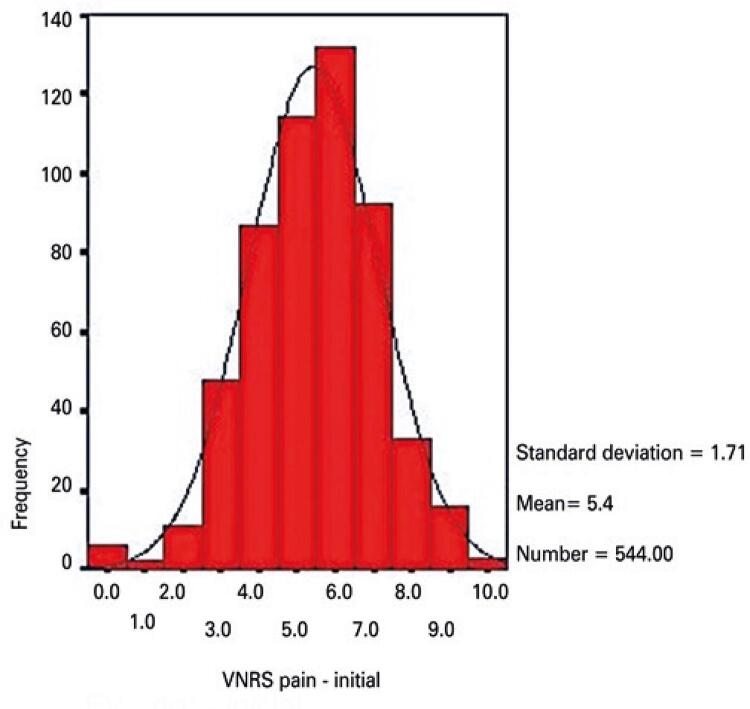
Histogram of frequencies of pain data at the start of physical therapy sessions

**Figure 4:**
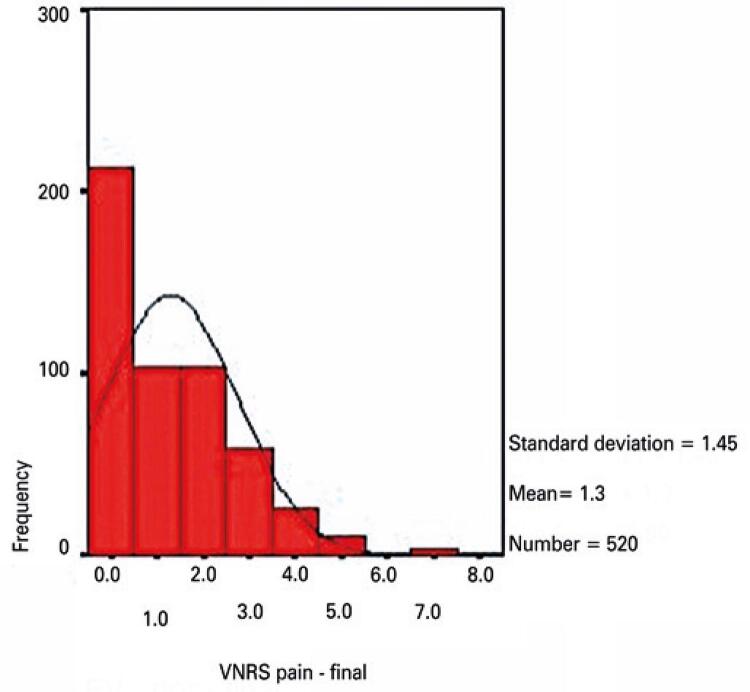
Histogram of frequencies of pain data at the end of physical therapy sessions

Initial and final pain intensity scores were compared to investigate whether physical therapy interventions were able to alleviate pain in the short term. Initial pain intensity data were symmetrically distributed, as revealed by histogram analysis (Figure 3). In turn, final pain intensity scores were asymmetrically distributed, with data skewed to the left (Figure 4). Hence, a non-parametric test for paired samples (Wilcoxon signed rank test) was selected. Differences were statistically significant (p=0.001).

VNRS: Verbal Numerical Rating Scale.

VNRS: Verbal Numerical Rating Scale.

**Figure 5:**
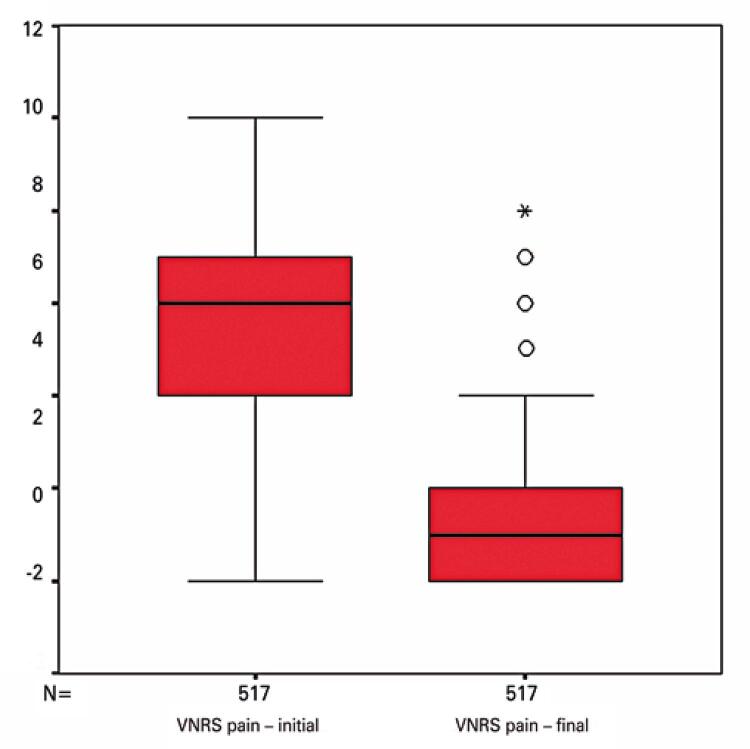
Box plot comparing variables initial and final pain intensity scores

Figure 5 shows the box plot depicting medians and interquartile ranges of initial and final pain intensity data clusters for visual comparative analysis of this variable.

Zero-to-ten scale where ten designates the worst imaginable pain (vertical axis). The box shows the first quartile (Q1) in the lower line, the median (Q2) in the central line and the third quartile in the upper line. Circles and stars indicate outliers.

VNRS: Verbal Numerical Rating Scale.

**Figure 6:**
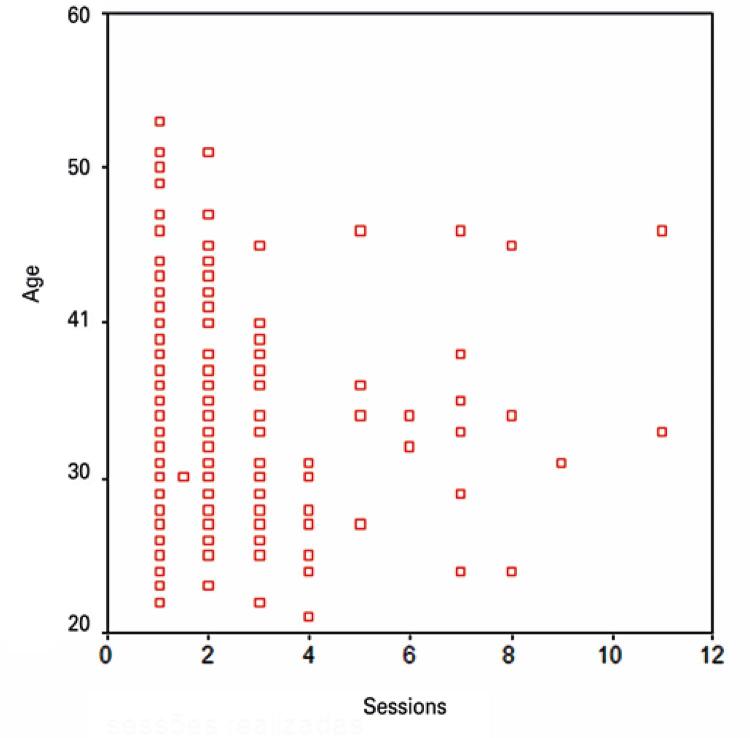
Scatter plot depicting the relation between age and number of physical therapy sessions

The age of military workers in this sample varied widely (Table 1). Therefore, correlations between this variable and the need of physical therapy care (*i.e*., number of sessions) were investigated. The variable “age” was asymmetrically distributed. Hence a non-parametric test (Spearman coefficient) was selected for linear correlation analysis. This test yielded a value of -0.077 and a corresponding two-tailed p value of 0.189, indicating a lack of correlation between age and number of physical therapy sessions. Figure 6 shows a scatter plot used for visual analysis of these findings.

## DISCUSSION

Health care professionals, including physical therapists, play an important role in the aftermath of catastrophes and environmental disasters, in spite of incipient definition.^(8)^ However, care is almost exclusively aimed at victims.^(7,9-11)^ This study revealed a paucity of scientific literature addressing the role of physical therapists in support provision to first-line professionals assigned to this type of operation.

This study describes a unique experience with regard to *in loco* provision of physical therapy care right after exposure to highly demanding activities by military firefighters involved in Brumadinho operations.

The expressive number of patients and physical therapy sessions suggest a high demand for physical therapy services, a sign of strong adherence to the therapeutic proposal implemented. In scenarios other than catastrophes involving military firefighters, physical therapy was shown to be effective and to encourage earlier resumption of professional activities compared to conventional medical treatment.^(12)^

The number of sessions per military worker varied. Age was regarded as a quantitative factor potentially related to physical therapy treatment seeking behavior. However, this hypothesis was rejected in statistical analysis. Other explanations for variations in treatment seeking behavior include individual work schedules, given workers spontaneously sought services while in mission, as well as the organization of work shifts. Since military workers were divided into teams working and resting away from the disaster area every 7 days, alternating schedules may have provided different opportunities of access to physical therapy services, with potential impacts on the number of sessions per military worker.

Most military workers receiving physical therapy care in this sample were members of CBMMG. This was not surprising, given the disaster site. The outstanding support provided by CBMMG was widely acknowledged throughout the country and the object of long-lasting media attention.

Most military workers seeking physical therapy services in this sample were male. This finding reflects the professional profile associated with the Military Firefighting institution, which employs only 10% of females. The predominance of males in this professional category explains the higher number of physical therapy interventions involving males in this sample. As an aside, literature data show that female military officers are more prone to musculoskeletal injuries.^(13)^

Physical therapy services were more commonly sought after by privates (soldiers, corporals, and sergeants). This may have reflected the larger number of privates relative to officers in the corporation and the nature of primary activities (search and rescue) performed by most military workers receiving physical therapy care. This type of “task-specific” ergonomic overload has been extensively reported.^(12,14,15)^

Firefighters are thought to have a 3.8 higher chance of sustaining musculoskeletal injuries,^(12)^ which are thought to be their primary occupational disorder.^(12,15)^ However, most military workers receiving physical therapy care in this sample failed to report prior injuries. Such retrospective data are extremely important for physical therapists, since they allow the determination of injury duration, as well as of potential causal or recurrence mechanisms. Findings of this study suggest a predominance of acute conditions, possibly due to the overload associated with the nature of work activities performed, as indicated by initial pain intensity scores (mean VNRS score, 5.43).

Spinal and generalized muscle and myofascial pain prevailed in this sample. This may also have reflected the nature of work activities performed. Atypical postures required to reach difficult to access areas or work on unstable surfaces, overload of specific muscle groups by repetitive gestures and the weight of uniforms and equipment^(12)^ contributed to increased energy expenditure, and may have led to muscle fatigue and tissue overload, with high risk of injury. Back pain is the most common cause of absenteeism and early retirement in this profession.^(16,17)^

Lower pain intensity at the end of physical therapy sessions as per the VNRS indicates that the goal of alleviating pain in the short term was fulfilled. This may have additional benefits, such as improved function, work performance, and psychological aspects.^(18)^ However, these variables were not investigated in this study.

The profile delineated in the literature (musculoskeletal involvement primarily in the spine, due to specific, work-related ergonomic issues) is consistent with findings of this study. Likewise, the adherence to and resolutive capacity of physical therapy treatment were confirmed, as shown by the expressive number of appointments and their positive impact on VNRS scores.

Methodological limitations of this study must be acknowledged, particularly with regard to pain relief findings. Given the lack of a Control Group, analgesic effects achieved cannot be attributed to physical therapy alone, since positive expectation and placebo effects were not controlled for. Other confounding variables, such as the Hawthorne effect (*i.e*., change in people’s behavior when they know they are part of an experiment), must also be accounted for in the analysis of findings presented.

## CONCLUSION

Physical therapists can provide significant support to teams involved in search and rescue operations in disaster settings. Physical therapy services implemented in Brumadinho were frequently sought after by military firefighters, and related interventions promoted immediate reduction in pain scores. Future controlled, randomized studies are warranted to confirm the efficacy of such interventions. Given the pioneer nature of this study, it may be used to inform the design of similar interventions and to support the role of physical therapists as essential players in the provision of care to operational teams in catastrophe scenarios.
